# Bioengineered extracellular vesicles: future of precision medicine for sepsis

**DOI:** 10.1186/s40635-023-00491-w

**Published:** 2023-03-10

**Authors:** Aina Areny-Balagueró, Anna Solé-Porta, Marta Camprubí-Rimblas, Elena Campaña-Duel, Adrián Ceccato, Anna Roig, Daniel Closa, Antonio Artigas

**Affiliations:** 1 Institut d’Investigació i Innovació Parc Taulí (I3PT), Parc Taulí Hospital Universitari, 08208 Sabadell, Spain; 2 Universitat Autònoma de Barcelona, 08193 Bellaterra, Spain; 3 Institut de Ciència de Materials de Barcelona, ICMAB-CSIC, Campus UAB, 08193 Bellaterra, Spain; 4 Present Address: Centro de Investigaciones Biomédicas en Red de Enfermedades Respiratorias, CIBERES-Instituto De Salud Carlos III, 28029 Madrid, Spain; 5 Servei de Medicina Intensiva, Corporació Sanitària i Universitària Parc Taulí, 08208 Sabadell, Spain; 6 Institut d’Investigacions Biomèdiques de Barcelona, Consejo Superior de Investigaciones Científicas (IIBB-CSIC), 08036 Barcelona, Spain

## Abstract

Sepsis is a syndromic response to infection and is frequently a final common pathway to death from many infectious diseases worldwide. The complexity and high heterogeneity of sepsis hinder the possibility to treat all patients with the same protocol, requiring personalized management. The versatility of extracellular vesicles (EVs) and their contribution to sepsis progression bring along promises for one-to-one tailoring sepsis treatment and diagnosis. In this article, we critically review the endogenous role of EVs in sepsis progression and how current advancements have improved EVs-based therapies toward their translational future clinical application, with innovative strategies to enhance EVs effect. More complex approaches, including hybrid and fully synthetic nanocarriers that mimic EVs, are also discussed. Several pre-clinical and clinical studies are examined through the review to offer a general outlook of the current and future perspectives of EV-based sepsis diagnosis and treatment.

## Introduction

Sepsis is an aberrant or dysregulated immune response to infection that leads to life-threatening organ dysfunction [1]. It is a highly prevalent condition that accounts for 18% of admissions to the intensive care unit (ICU) [2] and it is associated with a mortality rate higher than 25–30%, and even 40–50% when shock is present [3]. Although the incidence of sepsis is difficult to ascertain, conservative estimates indicate that sepsis is a leading cause of mortality and critical illness worldwide, accounted for almost 20% of all global deaths [4]. In addition, there is increasing awareness that patients who survive sepsis often have a reduced quality of life characterized by enduring cognitive and functional limitations [5, 6] turning survivorship into a public health problem with huge implications for patients, families, and the health care system.

The sepsis pathophysiology can be understood as a complex crosslinking of mechanisms, including inflammatory and anti-inflammatory responses, coagulopathies, systemic action of microorganisms, and multiple organ failure [7]. Such pathophysiology may substantially differ based on the underlying type of infection and individual host responses. In fact, a retrospective analysis using data from 63,858 patients allowed the identification and validation of four clinical sepsis phenotypes (α, β, γ, and δ) that were shown to correlate host-response patterns and clinical outcomes [8]. The high heterogeneity and the complex pathobiology of sepsis are the main reasons why pharmacological therapies are limited in preventing, managing, and diagnosing this syndrome, making it a “prototype” of a personalized treatment disease [9, 10].

Precision medicine aims to provide clinical treatments targeted to the needs of individual patients by considering their genetics, lifestyle and environment characteristics [11]. Regarding sepsis, precision medicine creates an individual approach on a case-by-case basis by identifying subgroups of patients with a high risk of adverse outcomes who may benefit from specific treatments or rescue therapies according to their particular phenotype [12]. In this regard, extracellular vesicles (EVs) bring along promises for tailoring sepsis treatment and diagnosis [13], since they possess several potential advantages compared with the cell therapies used for sepsis treatment, mainly addressing the inherent risks associated with live-cell transplants [14].

EVs, including exosomes, microvesicles and apoptotic bodies, are nanoscale (40–5000 nm size) phospholipid bilayer structures endogenously secreted from all cell types [15] and released in many body fluids such as urine, saliva, plasma, breast milk or cerebrospinal fluid [16]. Since EVs' discovery, research and methodological developments have led researchers to realize that they play crucial roles in cell-to-cell communications, the regulation of homeostatic and pathological processes, transferring proteins, bioactive lipid material, DNAs, RNA species as well as other cytoplasmic components [17]. The high biocompatibility and low immunogenicity, increased specificity to target cells or tissues, ability to cross biological barriers and use endogenous cellular machinery of loading, are features that make EVs an optimal candidate as drug delivery vehicles [18]. Moreover, EVs' composition depends on their generation pathway and the cells from which they originate. This confers them the capacity to provide evidence for early disease diagnosis and prognosis, disease severity evaluation, and treatment monitoring [19], making them a potential innovative approach for precision medicine.

The purpose of this article is to critically analyze the endogenous role of EVs in sepsis progression and highlight their use as diagnostic biomarkers and therapeutic agents for sepsis. We evaluate current progress and discuss future methods for EVs bioengineering to either boost the therapeutic effect that EVs show by themselves or how to use their  characteristics as drug delivery vehicles. More complex approaches, including hybrid and fully synthetic nanocarriers that mimic natural EVs are also discussed. Throughout the review, relevant pre-clinical and clinical studies are presented, which are so far encouraging towards a near future use of EVs for a more accurate and precise treatment for sepsis.

## Role of EVs in sepsis progression

Multiple pre-clinical and clinical studies of sepsis have reported increased numbers of circulating EVs in septic individuals [20, 21], and even higher levels in patients with septic shock, establishing a directly proportional relationship between the amount of EVs in plasma and the severity of the illness. Besides, circulating EVs have been associated with organ failure and mortality in critically ill sepsis patients [22]. Despite the EVs origin, whether from endothelial, epithelial, or immune cells, they all exhibit significant pro-inflammatory, pro-coagulant, and pro-permeability effects, influencing the behavior of EVs-targeting cells, which may contribute to the progression of the disease [23, 24]. Furthermore, circulating EVs populations and its content change during the several sepsis stages, which could be used to determine more precisely the severity and the pathology of each patient and, therefore, offer the possibility of a more precise intervention and therapy [21].

One of the most described actions of EVs during sepsis development is their pro-coagulant effect. Bacterial infections are associated with the release of EVs carrying tissue factor (TF, a significant initiator of the extrinsic coagulation cascade) by monocytes, which results in the activation of the coagulation pathway [24]. A recent study using a mouse model of pyroptosis showed that inflammasome activation leads to the release of TF-positive EVs into the blood, which in turn triggered blood coagulation, resulting in tissue perfusion deficit, organ dysfunction and lethality [27]. In addition, Wang et al. proposed TF-positive EVs as a biomarker for thrombosis risk in a mouse model of endotoxemia [28].

Another critical process during sepsis in which the EVs are also involved is the massive cytokine storm, which refers to multiple activated cascades that lead to an autoamplifying cytokine production, based on a profound increase in pro-inflammatory cytokines such as IL-1, IL-12, IL-18, tumor necrosis factor alpha (TNF-α), chemokines (IL8) and interferons into the circulation causing severe inflammation and tissue damage [29, 30]. Several studies have reported that some of the circulating cytokines are carried by EVs together with other chemokines and growth factors [31]. EVs from plasma of septic mice were shown to have the capability to enhance Th1/Th2 differentiation, promote T cells proliferation and augment T lymphocyte migration [31]. More specifically, EVs from pre-stimulated human neutrophils could act as attractors of monocytes via MCP-1 in vitro [32]. In addition, the kinetics of soluble or EV-associated cytokines and chemokines display different dynamics in the blood of LPS-injected mice. While the peak of soluble cytokines release is 2–12 h post-infection, the maximum of EVs carrying them is 12–24 h post-infection [31]. This, together with the fact that EVs exhibit an increased stability in the blood (allowing them to travel long distances within the body) [33], could suggest that EVs carrying pro-inflammatory cytokines is crucial for a sustained systemic inflammation over time.

EVs that are released during systemic inflammatory conditions can also contain DAMPs [34], including histones [35], heat shock proteins (HSPs) [36], and high-motility group box-1 (HMGB1) [37]. These proteins can interact with TLR4 or RAGE receptors, participating in the induction of different inflammatory pathways [34, 35]. C-reactive protein (CRP) is an acute phase protein that is part of the innate immune system and it is crucial for the activation of the adaptative immune response. It is predominantly secreted in response to tissue damage and systemic inflammatory conditions [38], and it is used for sepsis prognostic [39]. Indeed, Fendl et al. observed that the level of EVs containing CRP was significantly higher in septic patients than in healthy donors [40]. Similar results were observed in plasma EVs from moderate and severe acute pancreatic patients [41]. Both studies associated the presence of EVs carrying CRP with a major disease severity [40, 41].

Aside from proteins that directly trigger the transduction of signals, there are other EVs components like miRNAs capable of regulating gene expression [42]. Recent studies have evidenced the presence of a different plasma-circulating miRNA expression profile from EVs between septic and healthy patients [43, 44], which has been associated with sepsis severity. Interestingly, there are some specific miRNAs with a > 1.5-fold increase in EVs from septic mice compared to EVs from sham-operated control mice, such as miR-126-3p, miR-122-5p, miR-146a-5p, miR-145-5p, miR-26a-5p, miR-150-5p, miR-222-3p and miR-181a-5p which are closely related to inflammation and innate immune response, mediating the cytokine production via TLR7-MyD88 and NFκB signaling [20].

Severe sepsis is a stage of disease progression that eventually involves the alteration of vascular permeability [45, 46] triggering acute pulmonary edema, severe hypoxia, and consequently the development of acute respiratory distress syndrome (ARDS) [47]. Interestingly, EVs from septic patients' plasma have demonstrated to exert a detrimental role in microvascular permeability [48]. Specifically, EVs may cause direct injury to the endothelium modulating adherent junctions, tight junctions, caveolar and cytoskeletal proteins disturbing nitric oxide homeostasis [49].

Overall, the importance of endogenous EVs in sepsis was further highlighted in a study by Essandoh et al. in which GW4869, a neutral sphingomyelinase inhibitor that partially blocks the release of EVs, was used to successfully reduce the number of EVs and pro-inflammatory cytokines emitted from lipopolysaccharide-stimulated macrophages. This EV reduction was correlated with decreased systemic inflammation and mortality in a cecal ligation and puncture (CLP) mouse model[50].

## EVs as diagnostic markers

In sepsis, early diagnosis is crucial, thus, one-quarter of septic patients receive inadequate treatment and a worse prognosis as a consequence of a delayed diagnosis [51]. The current diagnostic criteria for sepsis are based on non-specific clinical symptoms, which can also occur in a variety of other clinical conditions [52]. In addition, it is particularly challenging in high-risk groups, such as the elderly or infants, which often present with atypical symptoms and are at an increased risk for ARDS or other secondary complications [53]. At present, there are no valid and reliable biomarkers allowing an on-site diagnosis and the identification of high-risk septic patients [54]. Hence, the search for new diagnostic markers to accurately stratify the stages of sepsis and facilitate early diagnosis remains meaningful and needed [55].

Research of endogenous EVs and their specific roles in sepsis progression reveal numerous diagnostic capabilities in pre-clinical sepsis models, as well as in human patients [56]. As mentioned above, EVs plasma levels in general have been proposed as predictive biomarkers for organ failure and mortality rate of septic patients [22], as well as, specific miRNA expression profile in EVs from septic patients, that has been also associated with sepsis survival and disease stage [57]. More specifically, proteomic analyses of human blood EVs from septic patients have revealed the presence of SPTLC3 protein, which was negatively correlated with disease progression [58]. In addition, Dakhlallah et al. detected an increased number of plasma EVs containing an increased amount of DNA methyltransferases mRNAs in the septic shock cohort compared to critically ill, non-septic control and sepsis cohorts [21], offering an opportunity to more precisely and promptly intervene. Although these studies highlight the potential of EVs as a novel method to diagnose and monitor the progression of the disease, further studies are needed to implement these methods in the clinics.

## EVs for sepsis treatment

Given the diversity of functions of EVs in the context of sepsis, they are currently being investigated as potential diagnostic biomarkers and therapeutic targets to suppress their detrimental role in sepsis progression [23]. Furthermore, the attributes associated with EVs, such as low immunogenicity and toxicity, excellent biocompatibility, and natural ability to cross biological barriers [19], have spurred pre-clinical and clinical investigations of EVs as a natural therapeutic strategy for many diseases [59–63]. Among all cell types, mesenchymal stem cells (MSCs) are major candidates for cell therapy over the last decades [64]. Several ongoing clinical trials use MSCs for sepsis and ARDS have provided promising results [65–67]. Hence, there has been a marked increase in published pre-clinical studies using MSC-EVs in animals with organ injury or immune dysfunction since 2013 until now [68]. All these studies have reinforced the crucial role of EVs derived from MSCs in reducing pathogen replication [69], phagocytosis [70], immunity regulation [71–73], and the regeneration of injured tissues, which are essential aspects to treat sepsis and its consequent organ dysfunction [66, 67] in vitro and in vivo.

## Methods for the modification and enhancement of natural EVs effect

Despite all advantages and positive therapeutic outcomes that natural EVs have demonstrated to exert, their translation to the clinical field requires extensive multidisciplinary efforts. Yet, there are still many challenges to overcome, including a lack of scalable production methods, low reproducibility of isolation techniques, and high heterogeneity between batches [63]. However, research has shown that advancements in the EVs biomanufacturing process and bioengineering methods can potentially hurdle these obstacles [64], opening up new future opportunities for EVs-based precision nanomedicine [65].

EVs, as natural intracellular communicators, have been also proposed as vehicles for the delivery of both native and non-native molecules [74]. Compared with standard delivery methods, EVs have been shown to deliver functional cargo with decreased immune clearance [75], higher stability in circulation, enhanced drug efficacy while minimizing drug toxicity and off-target side effects [76]. In addition, their unique structure, made of a hydrophobic lipid bilayer and a hydrophilic core, allows for the loading of a multitude of different cargoes [15]. Two main approaches exist for EVs loading, which are described below at some length.

### Non-cell-based methods

**Fig. 1:**
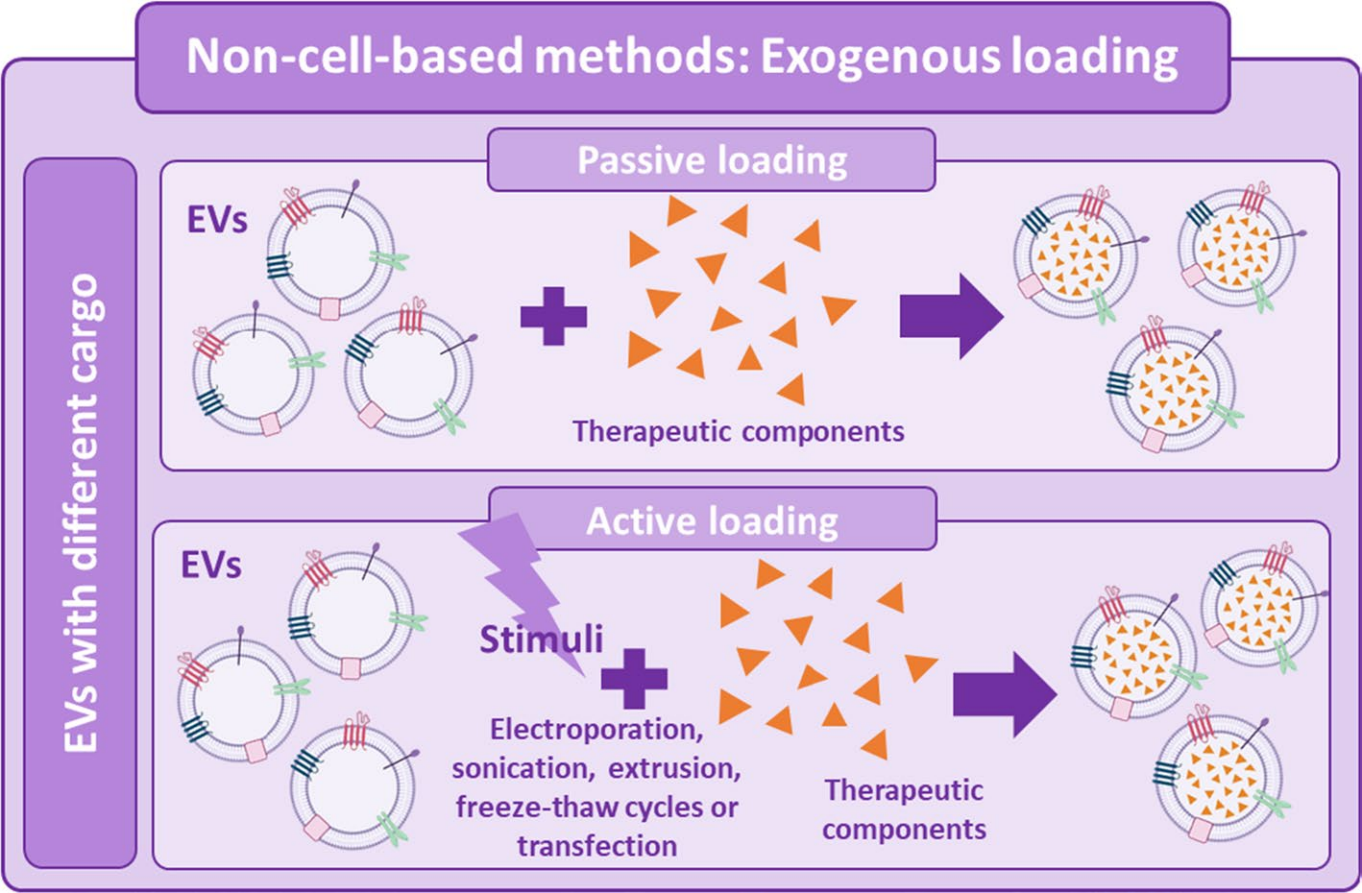
Non-cell-based methods for the modification of EVs cargo. *EVs* extracellular vesicles

On the one hand, non-cell-based methods, also known as exogenous loading [77] (Fig. 1), involves direct filling of already isolated EVs with therapeutic agents. This process can be accomplished by means of a passive encapsulation without using any external stimuli [78]. By this technique, the cargo can diffuse into the EVs following the concentration gradient [79], causing a lipid rearrangement of the membrane [80] or using energy-dependent channels [81]. Besides, exogenous loading can also be performed by an active encapsulation in which the EVs are forced to capture the desired cargo. Different routes for the loading process can be used, including electroporation, sonication, extrusion, freeze–thaw cycles and transfection [82]. While the passive method allows the preservation of EVs morphology [83], the active encapsulation shows a better loading efficiency and fewer difficulties in assessing the purity of the final preparation [84]. Figure 1 schematically describes two such approaches. Several studies have already proven drug loading feasibility into EVs and a higher efficiency of sepsis drugs when encapsulated within EVs. For instance, Sun et al. provided evidence that curcumin delivered by exosomes was more stable and highly concentrated in the blood. They demonstrated that curcumin carried by exosomes showed an enhanced anti-inflammatory effect in a lipopolysaccharide (LPS)-induced septic shock mouse model [80]. Two other relevant studies in this field showed a less disruptive EVs loading method involving a transmembrane pH gradient. Jeyaram et al. were able to load thousands of copies of miR-146a per EV and maintain their immunomodulatory properties, suggesting their potential therapeutic use in inflammatory diseases such as sepsis [85]. Similarly, in order to silence chemokine receptor 2 (CCR2), Ding et al. loaded EVs from mouse-derived immortalized bone marrow-derived macrophages (iBMDM) with siCCR2 by electroporation [86]. After intravenous administration, siCCR2 was delivered to the spleen and inhibited the infiltration of some inflammatory monocytes or macrophages in the spleen, alleviating the subsequent sepsis symptoms in a CLP mouse model [86]. In 2018, Gao et al. studied the usefulness of neutrophil-derived EVs as a delivery platform for piceatannol [87], an anti-inflammatory drug, described for its protective effect against sepsis-induced acute lung injury [88] and myocardial dysfunction [89]. They observed that piceatannol-loaded EVs dramatically alleviated acute lung inflammation/injury and sepsis in mice administered with LPS [87], revealing their potential application for precise nanomedicine.

### Cell-based methods

**Fig. 2:**
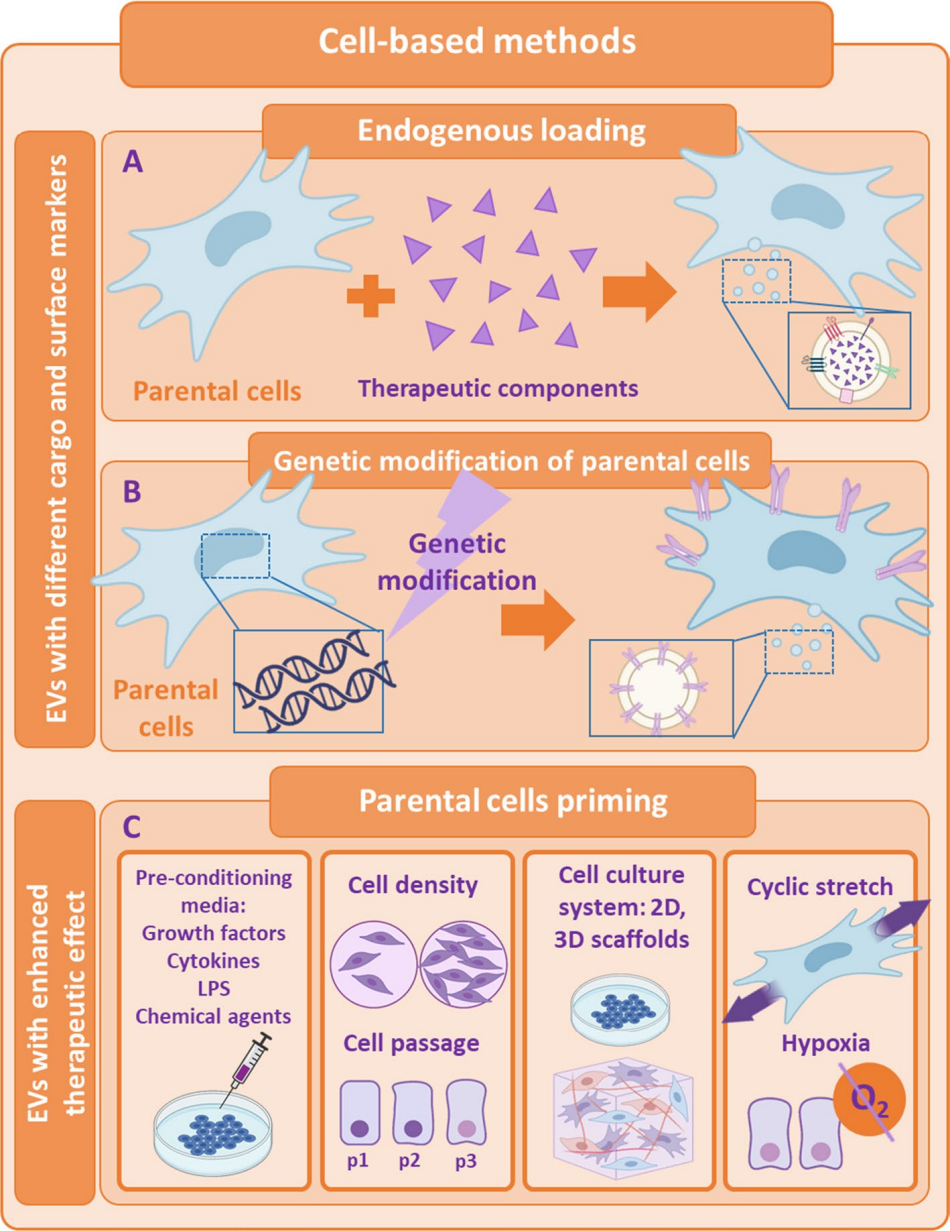
Cell-based methods for the modification of EVs cargo or surface markers and for the enhancement of their natural effect.** A** Endogenous loading;** B** Genetic modification of parental cells;** C** Priming of parental cells. *EVs* extracellular vesicles

On the other hand, there are cell-based strategies for filling EVs, also known as endogenous loading (see Fig. 2, panel A). In this approach, parenteral cells can be incubated with drugs or drug-loaded nanoparticles (NPs) [90], allowing the secretion of natural EVs carrying a certain fraction of the therapeutic content of interest. For example, Perteghella et al. developed a new system to obtain curcumin-loaded EVs by previously treating MSCs with NPs carrying curcumin. In the end, MSCs were able to release EVs entrapping curcumin nanoparticles [91], enhancing the loading efficiency in comparison with non-cell-based methods. Furthermore, parental cells can also be genetically or metabolically modified in order to alter and thus enhance their targeting ability and biocompatibility [83]. A very recent study focused on COVID-19 treatment, allowed the obtaining of genetically modified CAR-T cells to secrete programmed nanovesicles (NVs) that expressed on their surface two antibody single-chain fragment variables, which are demonstrated to block spike protein of SARS-CoV-2 binding to ACE2 receptor in vivo. Moreover, obtained NVs were loaded with an anti-viral drug, remdesivir, by electroporation. These innovative NVs prevented SARS-CoV-2 from entering cells expressing ACE2 and also inhibited intracellular virus replication redirecting specifically the NVs to the major sites of viral infection [92].

#### Enhancement of EVs' natural therapeutic effect

In addition to combating the obstacles that EVs as a therapy can present, there have also been described several strategies and progresses to improve the therapeutic natural effect exhibited by the EVs themselves [93]. EVs activity can be boosted by stress-induced adaptive responses of parental cells to the environment they are exposed to [94] (see Fig. 2, panel B). One of the most prominent strategy shown to significantly modify EVs intraluminal cargo, increase EVs secretion and enhance EVs potency is the alteration of parental cells cell-culture parameters [95, 96]. These modifications can be either biochemical (growth factors, cytokines, bacteria-derived molecules, pharmacological drugs or chemical agents) or biophysical (cell seeding density, cell-passages, 2D and 3D scaffolds, mechanical stimuli, etc.) [97, 98]. EVs secreted by primed MSCs have been extensively studied as a therapy for sepsis [99]. Increasing evidence indicate that EVs derived from IL-1β-primed MSCs induce more effectively M2-like polarization of macrophages, ameliorate the septic symptoms and increase the survival rate when administered in a CLP septic model. These findings are attributed to the appearance of exosomal miR-146a [100] and miR-21 [101], which are significantly upregulated in EVs from primed MSCs. Ti et al. reported similar results, although in their study, the MSCs-derived EVs with improved wound healing abilities and resolution of inflammation in a mouse model of diabetic cutaneous wound were obtained by pre-treating the MSCs with LPS, which, in this case, augmented the expression of exosomal let-7b [102]. Furthermore, inflammatory priming of MSCs with TNF-α or IFN-γ was also shown to trigger the secretion of EVs that were able to initiate the production of immunomodulatory factors, reducing Th1-cell proliferation [103] and inducing T-reg cell differentiation in vitro [104]. In this line, hypoxic preconditioning of MSCs seems to show akin effects since their secreted EVs also regulate inflammatory responses [105], inhibit apoptosis [106] and stimulate cellular proliferation [107] in different pre-clinical models by mainly modifying the miRNA expression profile from EVs, even though there are some studies that demonstrate the opposite [108].

The biophysical environment of MSCs can also modulate their secretory profile [109], and specifically, the EVs production [110]. MSCs that were cultured in a platform incorporating both physiomimetic lung extracellular matrix conditions and mechanical stimulation by cyclic stretch fostered their anti-inflammatory and immunosuppressive paracrine action in vitro [111] (see Fig. 2, panel C). Regarding the specific influence of physical atmosphere on EVs secretion and their immunomodulatory effect on sepsis, remains unclear. Even so, there are some studies that found a greater amount of protein, better outcomes in immunomodulation and vascularization in EVs isolated from MSCs seeded in 3D cultures [112], submitted to a cyclic mechanical stretch [113] or maintaining a low cell density and using only low passages for EVs collection [110] when administered in experimental models.

## Bioinspired synthetic EVs

**Fig. 3:**
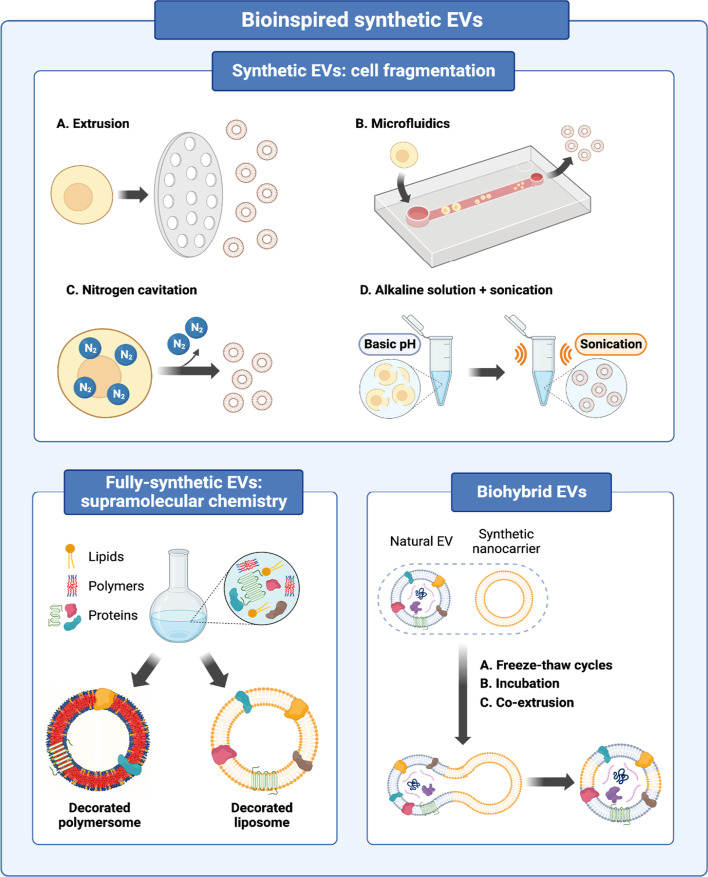
Different approaches to obtain bioinspired synthetic EVs. Strategies based on cell fragmentation, supramolecular chemistry and biohybrid EVs

Bioinspired synthetic EVs are artificially produced EVs that mimic their natural counterparts. They are being extensively studied, since they can overcome the cumbersome production of natural EVs, their tedious isolation, and other already-mentioned barriers that hinder their fast clinical translation [114]. Bioinspired synthetic EVs can be bioengineered via different strategies. They can be produced by cell fragmentation, using supramolecular chemistry, or fusing exosomes with synthetic nanomaterials to produce biohybrid structures [115]. These different approaches and examples are described below with more detail and are summarized in Fig. 3 and Table 1.

**Table 1:** Summary of bioinspired synthetic EVs generated by cell fragmentation, supramolecular chemistry or biohybrid approaches, and inorganic NPs used in the context of sepsis

Starting material	Preparation method	Cargo	Particle characterization	Biological effect	Refs.
Bioinspired synthetic EVs					
Human promyelocytic leukemia HL-60 cells	Cell fragmentation: nitrogen cavitation + differential UC	TPCA-1	Size: ~ 200 nm (cryo-TEM) ζ-potential: − 16 mV	Reduction of lung inflammation	[122]
Human monocyte U397 cells	Cell fragmentation: alkaline solution + sonication + UC	Dexamethasone	Size: 130 nm (DLS)	Reduction of IL-8 levels and mitigation of SIRS	[121]
MSCs	Cell fragmentation: serial extrusion (10, 5 and 1 μm) + DGUC	–	Size: 50–150 nm (TEM)	Decrease of inflammatory cytokines levels and immunomodulatory effect	[124]
Polymers (HA, PEI)	Supramolecular chemistry: polymer coupling and self-assembly	miR-7651-5p, miR-615-5p, miR-6239, miR-690, miR-206-3p, miR-466i-5p and miR-146a-5p	Size: ~ 100 nm	Reduction of TNF-α and IL-6 levels and relief of LPS-induced sepsis	[127]
Lipids (DPPC, DOPC, cholesterol) and membrane proteins from leukocytes	Supramolecular chemistry: mixing and assembly	–	Size: 94 nm (DLS) ζ-potential: − 27 mV	Decrease of IL-6, IL-1b and TNF-α, rise of IL-10 and TGF-β, and prolongation of murine survival	[128]
Amphotericin B-loaded liposomes	Commercial product	Amphotericin B	Size: 103 nm (DLS) ζ-potential: − 54 mV	Identification of sepsis biomarkers for diagnosis purposes	[129]
Polymer (Biotin-PEG-b-PAE(-g-PEG-b-DSPE)-b-PEG-Biotin)	Supramolecular chemistry: polymerization and self-assembly	Ciprofloxacin and TPCA-1	Size: 120 nm at pH = 7.4 (DLS)	Decrease in the number of leukocytes, bacteria and inflammatory cytokines, and mitigation of systemic inflammation	[130]
Macrophage membrane + lipids (PC and DSPE-PEG2000)	Biohybrid strategy: stirring + sonication + extrusion (800, 400 and 200 nm)	–	Size: 203 nm (DLS) ζ-potential: − 23 mV	Elimination of LPS and reduction of IL-1β, IL-6 and TNF-α levels	[134]
Inorganic NPs					
Fe_3_O_4_ NPs, chlorin e6 and aptamer	Fe_3_O_4_ NPs synthesis: thermal decomposition; EDC/NHS covalent coupling between Fe_3_O_4_ NPs, chlorin e6 and aptamer	–	Size: 17 nm (TEM) ζ-potential: − 10 mV	Detection, death and separation of bacteria from contaminated blood	[136]
Ceria NPs with mPEG-TK-PLGA coating	Ceria NPs synthesis: sol–gel; emulsion for polymer conjugation	Atorvastatin	Size: 43 nm (DLS) ζ-potential: − 4 mV	Suppression of excessive ROS levels in mitochondria and reduction of inflammation	[137]

*EDC* 1-ethyl-3-(3-dimethylaminopropyl)carbodiimide, *DLS* dynamic light scattering, *DGUC* density gradient ultracentrifugation, *DOPC* 1,2-dioleoyl-sn-glycero-3-phosphocholine, *DPPC* 1,2-dipalmitoyl-sn-glycero-3-phosphocholine, *DSPE* 1,2-distearoyl-sn-glycero-3-phosphorylethanolamine, *HA* hyaluronic acid, *LPS* lipopolysaccharide, *MSCs* mesenchymal stem cells, *NHS* N-hydroxysuccinimide, *PC* phosphatidylcholine, *PEG* polyethylene glycol, *PEI* polyethylenimine, *ROS* reactive oxygen species, *SIRS* systemic inflammatory response syndrome, *TK* thioketal, *TPCA-1* 2-[(aminocarbonyl)amino]-5-(4-fluorophenyl)-3-thiophenecarboxamide, *UC* ultracentrifugation

### Bioinspired synthetic EVs prepared by cell fragmentation

In the case of cell fragmentation, cells are forced to disintegrate to form nanosized vesicles. Their membranes contain natural lipids, proteins and nucleic acids from their parent cells, so they are very similar to natural EVs. Several fragmentation approaches have been reported to produce synthetic EVs starting from parental cells. Extrusion through nanosized polycarbonate membrane filters is widely used to turn cells into vesicles with reduced size, which preserve the topology of membrane proteins [116]. In addition, microfluidic systems have been developed to isolate, detect, analyze and engineer nanosized particles, such as exosomes [117, 118]. In these systems, living cells are forced to go through an array of parallel hydrophilic microchannels, in which they are broken into smaller fragments which then self-assemble forming artificial EVs [119]. Cells can also be disrupted by nitrogen cavitation to form exosome-like nanovesicles. This procedure relies on the dissolution of nitrogen in cells' cytoplasm under high pressure and a subsequent depressurization and release of this gas, causing the appearance of bubbles and the rupture of the cell membrane [120]. The exposure of cells to an alkaline solution forces the cells to break into membrane fragments, which can be re-assembled by sonication forming EVs.

One of the main advantages of cell fragmentation is the obtaining of homogeneous EVs populations. In addition, this approach is done in few steps, does not use organic solvents and can increase the production yield by more than 200-fold with respect to natural EVs [121], demonstrating the scalability of the method. Their physical and chemical compositions are not compromised, but the purification methods are still time-consuming.

Gao et al. successfully produced neutrophil-derived nanovesicles by two cycles of nitrogen cavitation under a pressure of 350–400 psi for 20 min, which triggered the physical disruption of neutrophils [122]. These neutrophil-derived EVs possessed integrin β_2_, a protein that binds to intercellular adhesion molecule 1 (ICAM-1), which is highly expressed in endothelial cells during inflammation [123]. These EVs were loaded with TPCA-1 (2-[(aminocarbonyl)amino]-5-(4-fluorophenyl)-3-thiophenecarboxamide) by incubation and were administered intravenously in an LPS model, reducing the lung inflammation and edema [122].

Furthermore, Go et al. developed EV-mimetic nanovesicles using human monocyte U397 cells, which were broken into membrane fragments by an alkaline solution and luminal cytosolic components were discarded by ultracentrifugation [121]. Sonication allowed the formation of EVs and their loading with dexamethasone, an anti-inflammatory agent. Using dexamethasone-loaded EVs, IL-8 levels in human umbilical vein endothelial cells (HUVECs) were reduced and systemic inflammatory response syndrome (SIRS) was mitigated in mice after intravenous administration of the nanovesicles loaded with the anti-inflammatory drug [121].

Park et al. produced EVs from MSCs by fragmenting cells through serial extrusions using different pore-sized polycarbonate membrane filters (10, 5 and 1 µm) [124]. The administration of MSC-derived EVs significantly decreased cytokine release into systemic circulation and monocyte and neutrophil infiltration in the peritoneum, demonstrating the immunomodulatory effect of MSC-derived EVs in a murine model of sepsis [124].

### Bioinspired fully synthetic EVs prepared by supramolecular chemistry

Bioinspired artificial EVs can be produced by bottom-up approaches using individual components of the cellular membrane (lipids and proteins), which interact by supramolecular chemistry to form spherical structures that mimic EVs [115]. These nanocarriers show great potential as exosome-mimics when they are modified with chemical groups or conjugated with specific biomolecules, such as membrane proteins, peptides, or antibodies, to simulate the composition of natural EVs. These bottom-up approaches need a complete understanding of each component in the naturally derived exosomes to develop nanocarriers with superior characterization control and clean composition [116]. These entirely synthetic EVs may better satisfy the specifications of medical regulative agencies and have higher pharmaceutical acceptability than their natural counterparts because of their homogeneous and reproducible production, and besides, their cargoes can be loaded more efficiently. Nevertheless, further research is required to reproduce a complex lipid and protein natural structure starting from the building blocks. Liposomes, with their lipid double layer, and polymerosomes have emerged as strategic elements in cell mimicking [125]. Furthermore, biodegradable polymers, such as poly(lactic-*co*-glycolic acid) (PLGA), have also been used to develop nanocarriers that resemble EVs in size and morphology. PLGA nanocarriers have been proposed as a potential pulmonary drug delivery system, since they did not exhibit signs of cytotoxicity and showed and excellent lung biodistribution after intratracheal instillation, offering a suitable tool for the treatment of sepsis-induced acute lung injury [126]. Below, several studies are shown that demonstrate the efficacy of this type of nanocarriers for the treatment of sepsis-induced ARDS.

Li et al. synthesized hyaluronic acid-polyethylenimine (HA-PEI) NPs to mimic exosomes secreted by tumor cells that were shown to exert protective effects against sepsis [127]. This process included the chemical coupling of HA and PEI and the self-assembly of the conjugated polymer into nanosized particles, which were loaded with the seven miRNAs that were identified as the responsible for the therapeutic effect of tumoral exosomes. They observed that the obtained NPs did not alter either cell viability or permeability, and they were protective against inflammation [127]. Moreover, serum levels of cytokines TNF-α and IL-6 were decreased, and LPS-induced sepsis in mice and cynomolgus monkeys was relieved after the administration of these NPs, being promising candidates for the treatment of sepsis and cytokine-storm-related conditions [127].

Molinaro et al. engineered liposome-like nanocarriers, namely leukosomes, by blending synthetic lipids (1,2-dipalmitoyl-sn-glycero-3-phosphocholine (DPPC), 1,2-dioleoyl-sn-glycero-3-phosphocholine (DOPC) and cholesterol at 4:3:3 molar ratio) with membrane proteins from leukocytes [128]. In vitro studies with macrophages treated with leukosomes demonstrated a decreased expression of pro-inflammatory genes (IL-6, IL-1b, and TNF-α) and a raised expression of anti-inflammatory ones (IL-10 and TGF-β), indirectly causing an anti-inflammatory response [128]. In vivo experiments in an LPS-induced model of sepsis elucidated that leukosomes allowed the targeting of inflamed tissues and significantly prolonged survival [128].

Papafilippou et al. used commercially available and clinically used amphotericin B-loaded liposomes (AmBisome^®^) to rapidly and accurately diagnose and differentiate between sepsis, triggered by an infection, and non-infectious acute systemic inflammation in humans [129]. After incubating these liposomes with plasma from two groups of patients, one suffering from sepsis and the other from a non-infectious acute systemic inflammation, a protein corona was formed through the spontaneous interaction of plasma proteins with liposomes in both cases [129]. The protein corona was deeply characterized and compared by mass spectrometry to demonstrate that the proposed synthetic nanosystem allowed the identification of 67 potential biomarker proteins for the reproducible distinction between non-infectious acute systemic inflammation and sepsis [129]. Therefore, the liposome-corona platform could fasten the clinical evaluation and precisely diagnose sepsis, avoiding unneeded antibiotic treatments.

Zhang et al. designed and synthesized polymeric micelles by self-assembly using an amphiphilic block copolymer (Biotin-PEG-b-PAE(-g-PEG-b-DSPE)-b-PEG-Biotin). The micelles were loaded with an antibiotic (ciprofloxacin) and an anti-inflammatory agent (TPCA-1) to prevent bacterial dissemination and mitigate inflammation [130]. In addition, the polymeric micelles were coated with ICAM-1 antibodies to target infected tissues, which enhanced drug delivery efficacy [130]. These nanosystems were responsive to the acidic pH and bacterial enzymes present in infectious microenvironments, triggering the release of the drugs [130]. The administration of the drug-loaded micelles in an acute peritonitis model significantly reduced the leukocytes, bacteria, and inflammatory cytokines, indicating a suppression of the peritoneal infection, leading to a mitigated systemic inflammation [130].

### Biohybrid EVs

Natural EVs and synthetic nanostructures, such as liposomes or polymersomes, can be fused to form biohybrid vesicles that combine the advantages of both systems without altering their intrinsic properties [115]. Synthetic nanosystems contribute with stability, high and controlled production and the possibility of drug loading. There are different methods by which biohybrid EVs can be produced, such as the freeze–thaw technique, in which natural EVs and liposomes are mixed, frozen and thawed for several cycles [131]. Another option is by simply incubating natural EVs and liposomes, which may lead to the formation of biohybrid structures without external stimuli, since both have a lipid bilayer [132]. Finally, membranes of exosomes and liposomes break and merge to form hybrid EVs by the co-extrusion method, in which they undergo physical stress when going through membrane pores [133].

Regarding the utilization of biohybrid EVs for sepsis treatment, Jiang et al. used sonication to develop a macrophage-mimetic hybrid liposome by fusing macrophage membranes with artificial lipids (phosphatidylcholine and DSPE-PEG2000), which stabilized the natural membrane and prolonged the blood circulation time [134]. The resulting macrophage-mimetic hybrid liposome combined the advantages of both natural and artificial membranes. It reduced the toxicity of LPS in activated macrophages, protecting mice against septic shock, and reducing the levels of IL-1β, IL-6 and TNF-α [134].

## Inorganic nanoparticles for sepsis

In addition to synthetic bioinspired EVs, several inorganic nanoparticles (NPs) have also been investigated for the diagnosis and treatment of sepsis to mimic the natural effect of EVs. The use of NPs for these purposes presents great potential due to the possibility of engineering NPs with different compositions, sizes, shapes, and surface charges and their capacity for surface functionalization, allowing targeting and selective binding [135]. Still, toxicity, rapid clearance, and difficulties crossing some biological barriers are these systems' main drawbacks [115]. However, magnetic NPs, metallic NPs or quantum dots, combined with lab-on-a-chip devices, point-of-care (POC) technologies or biosensors, have been examined for fast and sensitive sepsis detection and therapy [135].

As an example, Wang et al. developed a nanosystem for early sepsis diagnosis and extracorporeal blood disinfection as a treatment to eliminate the bacteria causing sepsis [136]. The platform was based on iron oxide (Fe_3_O_4_) NPs functionalized with chlorin e6 (Ce6) and bacterial species-identifiable aptamers (Apt) [136]. The aptamer helped to capture bacteria, while the Fe_3_O_4_ NPs allowed magnetic separation for the detection and enrichment of bacteria, and Ce6 acted as a photosensitizer that exhibited high photosensitizing efficacy. Based on this, the Fe_3_O_4_–Ce6–Apt nanosystem allowed a successful and rapid diagnosis of sepsis caused by single or multiple species of bacteria (*S. aureus* and *E. coli*) [136]. Due to the strong photodynamic effect and magnetic properties of the Fe_3_O_4_–Ce6–Apt nanosystem, it was also studied for the disinfection of extracted contaminated blood by first irradiating it with a NIR laser, causing the death of bacteria, and then magnetically removing the pathogens [136]. Disinfected blood could be reused for mice transfusion without adverse reactions, suggesting the successful potential of the Fe_3_O_4_–Ce6–Apt nanosystem for sepsis treatment [136]. In addition, Yu et al. designed and synthesized a reactive oxygen species (ROS)-responsive nanosystem which combined mitochondria-targeting ceria (CeO_2_) NPs with atorvastatin for acute kidney injury caused by sepsis [137]. CeO_2_ NPs were conjugated with triphenylphosphine, coated with mPEG-TK-PLGA, a ROS-responsive organic polymer that improved the biocompatibility of the nanosystem, and subsequently loaded with atorvastatin [137]. The NPs were accumulated in the kidneys and targeted specifically the mitochondria to suppress excessive ROS levels. They also efficiently reduced inflammation in vivo and exhibited antioxidant and antiapoptotic effects in vitro [137]*.*

## Conclusions

Sepsis is a complex syndrome characterized by its high heterogeneity between patients, which hinders its diagnosis and therefore, the administration of an efficient and definitive treatment. The need for a more personalized management of sepsis and the malleable characteristics of EVs, whether synthetic or natural, may be the focal point for the development of a therapy targeting all different pathways that confer its pathophysiology in the near future.

Here we have presented not only the role that EVs play in the progression of sepsis and how it could be used to develop more precise diagnosis and prognosis methods, but also highlighted the potential of natural EVs as a treatment for sepsis. In addition, the research discussed presently shows the wide range of emerging bioengineering strategies to enhance the beneficial effect of EVs or their use as cell-based delivery systems, which, in turn, leads to overcoming the inherent challenges that cellular therapy exerts. Furthermore, the recent research advancements in nanotechnology have opened up several exciting avenues to develop innovative approaches with a marked translational character, offering a potential precise therapy for sepsis.

Despite the promising prospects described in this review, future research is needed to further study the contribution of EVs in sepsis progression to provide a useful and prompt diagnostic method to determine more precisely the severity and the specific sepsis phenotype of each patient. Besides, additional investigation in regard to the septic pathological environment is required, as well as, the refinement of engineering processes and clinical development in order to obtain a definitive EVs-based sepsis treatment.

## Data Availability

Not applicable.
